# Late gadolinium enhancement in pulmonary hypertension predicts clinical events

**DOI:** 10.1186/1532-429X-14-S1-P86

**Published:** 2012-02-01

**Authors:** Siva Kumar Soma, Vishal Goyal, Vikas K Rathi, Mark Doyle, Srinivas Murali, Diane A Vido, Raymond Benza, George Sokos, Robert W Biederman

**Affiliations:** 1 Allegheny General Hospital, Pittsburgh, PA, USA; 2 Bon Secours Heart and Vascular Institute, Richmond, VA, USA

## Summary

Late Gadolinium Enhancement (LGE) has been shown to be a poor prognostic indicator in LV failure. However, it has not been studied in predicting adverse prognosis in Pulmonary Hypertension (PH). Herein we show that LGE enhancement predicts major adverse clinical events (MACE) in PH patients.

## Background

Introduction: Right ventricular (RV) function predicts prognosis in pulmonary hypertension (PH) patients (pts) and Right Ventricular (RV) failure. Prior studies evaluating of 3D RV ejection fraction (EF) have yielded inconsistent prognostic information. Here we explore the prognostic value of contrast enhanced CMR in PH (WHO group 1-5) patients.

### Hypothesis

We hypothesize that myocardial Late Gadolinium Enhancement (LGE), a marker for myocardial fibrosis when present in RV or RV insertion points (RVIP), is a predictor of adverse prognosis in PH pts.

## Methods

A retrospective chart review of PH pts (n=42) who underwent clinically indicated CMR were analyzed. Demographic data showed mean age 61 yrs; 26% male; 55% WHO group 1 21% group 2, 5% group 3, 14% group 4, 5% group 5. RV volumetric data were indexed to BSA, and along with RVIP LGE information, were correlated with major adverse clinical events (MACE) including hospitalization, death, referral/need for lung transplantation and need for addition/increase in inotropic therapy. In WHO group 2, lung transplantation was not a MACE event as it is not a relevant clinical option.

## Results

LGE was positive (+) in 18 pts (43%) and negative (-) in the remaining 24 pts (57%). The predominant MACE events occurs in the LGE+ group (78%). Specifically, in LGE+ group, 7 pts (39%) had MACE while 11 pts (61%) did not have MACE. In comparison, the LGE- group had only 2 pts (8%) who had MACE and 22 pts (92%) who did not have MACE, <0.03 for all). The results were similar when WHO group 1 were subanalyzed. In WHO 1 subgroup 11 pts (48%) were LGE+ and 12 pts (52%) were LGE-. In the LGE+ group 4pts (36%) had MACE while 7 pts (64% did not. In the LGE- there were no MACE, (<0.04). Fisher’s exact test was used for group comparisons. Univariate analysis revealed only RVESVI, RVEF, RVEDVI and MRI LGE predicts MACE. However, via multivariable logistic regression analysis only RVESVI (OR: 1.1, 95%; CI 1.0-1.2) and MRI LGE (OR: 7.0,95%;CI 1.2-39.5) predict MACE. (χ2=22.5,df=2,N=42, p<0.001).

## Conclusions

Late Gadolinium Enhancement is a seven-fold stronger predictor of MACE than standard CMR metrics. LGE's role as an independent adverse prognosticator may define the pathophysiologic hallmark in PH patients as a direct reflection of underlying RV failure due to progressive myocardial fibrosis.

## Funding

Internal.

